# Autistic symptoms in unaffected first-degree relatives of people with schizophrenia: results from the Italian Network for Research on Psychoses multicenter study

**DOI:** 10.1192/j.eurpsy.2023.2455

**Published:** 2023-10-23

**Authors:** Antonio Vita, Stefano Barlati, Giacomo Deste, Alessandro Rossi, Paola Rocca, Alessandro Bertolino, Eugenio Aguglia, Carlo A. Altamura, Mario Amore, Antonello Bellomo, Paola Bucci, Bernardo Carpiniello, Alessandro Cuomo, Liliana Dell’Osso, Luigi Giuliani, Carlo Marchesi, Giovanni Martinotti, Palmiero Monteleone, Cristiana Montemagni, Gabriele Nibbio, Massimo Pasquini, Maurizio Pompili, Antonio Rampino, Rita Roncone, Rodolfo Rossi, Alberto Siracusano, Elena Tenconi, Patrizia Zeppegno, Silvana Galderisi, Mario Maj, Italian Network for Research on Psychoses

**Affiliations:** 1 Psychiatric Unit, School of Medicine, University of Brescia, Brescia, Italy; 2 Department of Mental Health, Spedali Civili Hospital, Brescia, Italy; 3 Section of Psychiatry, Department of Biotechnological and Applied Clinical Sciences, University of L’Aquila, L’Aquila, Italy; 4 Department of Neuroscience, Section of Psychiatry, University of Turin, Turin, Italy; 5 Department of Neurological and Psychiatric Sciences, University of Bari, Bari, Italy; 6 Department of Clinical and Molecular Biomedicine, Psychiatry Unit, University of Catania, Catania, Italy; 7 Department of Psychiatry, University of Milan, Milan, Italy; 8 Section of Psychiatry, Department of Neurosciences, Rehabilitation, Ophthalmology, Genetics and Maternal and Child Health, University of Genoa, Genoa, Italy; 9 Psychiatry Unit, Department of Medical Sciences, University of Foggia, Foggia, Italy; 10 Department of Psychiatry, University of Campania “Luigi Vanvitelli”, Naples, Italy; 11 Section of Psychiatry, Department of Public Health, Clinical and Molecular Medicine, University of Cagliari, Cagliari, Italy; 12 Department of Molecular Medicine and Clinical Department of Mental Health, University of Siena, Siena, Italy; 13 Section of Psychiatry, Department of Clinical and Experimental Medicine, University of Pisa, Pisa, Italy; 14 Department of Neuroscience, Psychiatry Unit, University of Parma, Parma, Italy; 15 Department of Neuroscience and Imaging, G. d’Annunzio University, Chieti, Italy; 16 Department of Medicine, Surgery and Dentistry “Scuola Medica Salernitana”, University of Salerno, Salerno, Italy; 17 Department of Neurology and Psychiatry, Sapienza University of Rome, Rome, Italy; 18 Department of Neurosciences, Mental Health and Sensory Organs, S. Andrea Hospital, Sapienza University of Rome, Rome, Italy; 19 Unit of Psychiatry, Department of Life, Health and Environmental Sciences, University of L’Aquila, L’Aquila, Italy; 20 Department of Systems Medicine, Psychiatry and Clinical Psychology Unit, Tor Vergata University of Rome, Rome, Italy; 21 Psychiatric Clinic, Department of Neurosciences, University of Padua, Padua, Italy; 22 Department of Translational Medicine, Psychiatric Unit, University of Eastern Piedmont, Novara, Italy

**Keywords:** autism spectrum disorder, cognition, endophenotype, psychosocial functioning, schizophrenia first-degree relatives, social cognition

## Abstract

**Background:**

Autistic symptoms represent a frequent feature in schizophrenia spectrum disorders (SSD). However, the prevalence and the cognitive and functional correlates of autistic symptoms in unaffected first-degree relatives of people with SSD remain to be assessed.

**Methods:**

A total of 342 unaffected first-degree relatives related to 247 outpatients with schizophrenia were recruited as part of the multicenter study of the Italian Network for Research on Psychoses (NIRP). Autistic features were measured with the PANSS Autism Severity Scale. Three groups of participants, defined on the presence and severity of autistic symptoms, were compared on a wide array of cognitive and functional measures.

**Results:**

Of the total sample, 44.9% presented autistic symptoms; 22.8% showed moderate levels of autistic symptoms, which can be observed in the majority of people with SSD. Participants with higher levels of autistic symptoms showed worse performance on Working Memory (*p* = 0.014) and Social Cognition (*p* = 0.025) domains and in the Global Cognition composite score (*p* = 0.008), as well as worse on functional capacity (*p* = 0.001), global psychosocial functioning (*p* < 0.001), real-world interpersonal relationships (*p* < 0.001), participation in community activities (*p* = 0.017), and work skills (*p* = 0.006).

**Conclusions:**

A high prevalence of autistic symptoms was observed in first-degree relatives of people with SSD. Autistic symptoms severity showed a negative correlation with cognitive performance and functional outcomes also in this population and may represent a diagnostic and treatment target of considerable scientific and clinical interest in both patients and their first-degree relatives.

## Introduction

### Background

Schizophrenia spectrum disorders (SSD) and autism spectrum disorders (ASD) are two distinct nosological entities, characterized by different age of onset, course of the disorder, and treatment response [1, 2]. Psychotic symptoms do not represent an essential feature of ASD, and symptoms can generally be observed at a much earlier age: these distinctions as well as differences on a neurobiological level have led some researchers to hypothesize that SSD and ASD represent opposite neurodevelopmental models [3].

Notwithstanding these differences, SSD and ASD share several remarkable overlaps: impairment in social cognition abilities represents a core feature of both spectra, with very similar levels of limitation across the disorders [4–7]. Neurocognitive performance, particularly in the domains of processing speed, verbal comprehension, and working memory, appears to be similar in people diagnosed with SSD or ASD [8, 9].

Important similarities can be also observed at a genetic level and in neuroanatomical and neurofunctional imaging [10–15].

ASD symptoms are more frequent in people diagnosed with SSD than in healthy individuals [16–18]. Subjects with a childhood diagnosis of ASD are frequently diagnosed with SSD during adolescence and early adulthood [19–22], and 30% of young people receiving a diagnosis of very early-onset schizophrenia also present a concomitant diagnosis of ASD [23].

Autistic features also appear to have a significant impact on several cognitive, clinical, and functional outcomes in people living with SSD and have, therefore, recently become a topic of increasing scientific interest: autistic symptoms in people with SSD are related to worse social cognition performance and worse real-world functioning [24–28] and could represent a negative moderator of response to pharmacological treatment [29, 30] and psychosocial interventions such as social cognition-oriented cognitive remediation [31].

However, autistic symptoms may also have some protective effects: some studies report that autistic symptoms could mitigate the negative impact on functioning produced by high levels of psychotic symptoms [32–34]. Some studies also report that autistic symptoms appear to be related to other positive outcomes: one study has found a positive correlation between the severity of autistic symptoms and better stigma resistance, as measured by the Stigma Resistance factor of the Internalized Stigma of Mental Illness [35]; another study has reported that individuals diagnosed with SSD showing more prominent autistic features present better real-world social acceptability, as measured by the dedicated subscale of the Specific Level of Functioning Scale (SLOF), compared to other participants with same diagnosis with less severe autistic symptoms [28]. These effects may be partly explained by differences in relational and coping styles observed in individuals with prominent autistic features [36].

Despite this recent scientific and clinical interest in the role of autistic symptoms and the characteristics related to an autistic phenotype in SSD, their impacts on the lives of first-degree relatives of people diagnosed with SSD are currently scarcely explored.

SSD presents a considerable genetic component and a high degree of heritability [37, 38], and first-degree relatives of people living with SSD present a profile of neurocognitive and social cognition performance that is intermediate between unaffected controls and individuals diagnosed with SSD [39–42] and are usually considered an intermediate phenotype of SSD [43]. Pathways of real-world functional impairment are also similar in people living with SSD and their first-degree relatives [44].

The role of features related to an autistic phenotype in people living with SSD was investigated in a large sample of patients with schizophrenia included in the baseline multicenter study of the Italian Network for Research on Psychoses (NIRP) [45]. The study showed worse cognitive performance and worse real-world outcomes in several domains, but better real-world social acceptability in subjects with higher levels of autistic symptoms [28]. The NIRP study also included a large cohort of first-degree relatives of people with SSD [42], in which the prevalence of autistic symptoms and their role on real-world outcomes were not previously investigated. As people with a family history of SSD in general also present a consistently increased risk of ASD diagnosis [46, 47], exploring the prevalence and impact of autistic symptoms in first-degree relatives of people with SSD could provide valuable insight both in a scientific and in a clinical perspective.

### Aims

The aims of the present study were to assess the prevalence of autistic symptoms in first-degree relatives of people diagnosed with schizophrenia and to investigate their cognitive and functional correlates. In particular, the study compared participants without autistic symptoms, with minimal autistic symptoms, and with moderate autistic symptoms on demographic, neurocognitive, sociocognitive, and real-world functional measures. The main hypothesis of the study is that subjects with more severe levels of autistic symptoms and a more pronounced autistic phenotype would show worse cognitive performances and worse real-world outcomes compared to other participants.

## Methods

### Sample

For the present study, the database of first-degree relatives of people living with schizophrenia recruited in the NIRP was used.

The NIRP is a large research network involving 26 Italian university psychiatric clinics and mental health departments. Its database includes a sample of 921 people diagnosed with schizophrenia living in the community [44, 45].

For each recruited patient who agreed to involve relatives, two first-degree relatives were recruited, when available. They had to be, in order of preference, the two parents, or one parent and one sibling, or two siblings. Relatives were included in the study if they did not meet criteria for a current or lifetime psychiatric diagnosis as assessed by the Structured Clinical Interview for DSM-IV–Non-Patient version (SCID-I/NP) and the Structured Clinical Interview for DSM-IV axis II Disorders (SCID-II).

Further exclusion criteria were: (1) a history of head trauma with loss of consciousness, (2) neurological disease, (3) a history of alcoholism or substance abuse in the last 6 months, and (4) inability to provide informed consent.

Three hundred forty-two first-degree relatives (M:F = 145:197, age 53.9 ± 13.5 years, education 11.5 ± 3.9 years) related to 247 participants diagnosed with schizophrenia were recruited and completed the assessment and were included in the present analyses [42].

Participants were recruited from March 1, 2012, to September 30, 2013.

All included subjects provided written informed consent after receiving a comprehensive explanation of study procedures and goals. The study protocol was approved by the ethical committee of the coordinating center and of all other participating centers (approval number 73/2012).

### Assessment

The assessment was conducted within 2 weeks after subjects’ recruitment. According to the same procedure in all centers, enrolled participants completed the assessment for the study in 2 days: sociodemographic and clinical assessment on day 1 in the morning and assessments of neurocognitive functions, social cognition, and functional capacity on day 2 in the morning.

A complete description of study recruitment and assessment procedures, including inter-rater reliability and comparability of data collection procedures, has been reported elsewhere [28, 42, 44, 45].

### Autistic symptoms assessment

Autistic symptoms severity was assessed in all included participants using the PANSS Autism Severity Scale (PAUSS) [48]. The PAUSS is a scale composed of eight items ranging one to seven derived from the Positive and Negative Syndrome Scale [49] and has been designed specifically to assess the expression of an autistic phenotype in people with SSD. Included items feature N1 (“blunted affect”), N3 (“poor rapport”), N4 (“social withdrawal”), N5 (“difficulties in abstract thinking”), N6 (“lack of spontaneity and flow of conversation”), N7 (“stereotyped thinking”), G5 (“mannerism”), and G15 (“preoccupation”): these features, rather than assessing the presence and diagnosis of ASD as a distinct neurodevelopmental disorder, explore the severity of difficulties in social interactions and in communication and the limited, repetitive, and stereotypic patterns of behavior that characterize the autistic phenotype in people with SSD [48, 50, 51].

Its validity and precision have been already demonstrated and found to be satisfying, with the PAUSS strongly correlating with other more established diagnostic tools for the assessment of autistic features and showing even better sensitivity than such scales in measuring autistic symptoms severity in people with SSD [50, 52]. It has also been used to assess genetic and neurobiological correlates of autistic features in people with SSD [53–55] and in first-episode psychosis [32, 56, 57] as well as healthy subjects [58].

According to the original validation study cut-offs [48], the sample was divided into participants with no ASD symptoms (PAUSS = 8), minimal ASD symptoms (8 < PAUSS ≤ 10), and moderate ASD symptoms (PAUSS ≥ 11). This partitioning was structured considering that the investigated sample was composed of subjects without a diagnosis of SSD or ASD, so a high number of subjects without ASD symptoms and no subject with severe ASD symptoms (PAUSS ≥ 30) was expected.

### Cognitive assessment

Cognitive performance was assessed using the MATRICS Consensus Cognitive Battery (MCCB) [59]. The MCCB is a cognitive assessment battery with the highest level of recommendation for use in both clinical and research settings according to recent international guidance [60] and is composed of specific tasks assessing the following cognitive domains: speed of processing (Trail Making Test Part A; Brief Assessment of Cognition in Schizophrenia: Symbol Coding; Category Fluency Test: Animal Naming), verbal and spatial learning (Hopkins Verbal Learning Test–Revised, immediate recall; Brief Visuospatial Memory Test–Revised), reasoning and problem-solving (Neuropsychological Assessment Battery, Mazes subtest), attention (Continuous Performance Test: Identical Pairs), working memory (Wechsler Memory Scale, Spatial Span subset; Letter Number Span Test), and social cognition (Mayer-Salovey-Caruso Emotional Intelligence Test: Managing Emotion task). A *t*-score was computed for each cognitive domain, corrected for gender, age, and education, and a global cognitive composite score was finally calculated following the recommendation of the battery developers [61].

### Functional outcomes measures

Functional capacity was assessed with the UCSD Performance-Based Skills Assessment, Brief (UPSA-B) [62]. The UPSA-B is a brief and widely used performance-based instrument that assesses skills involved in community tasks: “financial skills” (e.g., counting money and paying bills) and “communication skills” (e.g., to dial a telephone number for emergency or reschedule an appointment by telephone), with a total score ranging from 0 to 100.

Global personal and social functioning was assessed with the Personal and Social Performance (PSP) scale [63]. The PSP in a single-item, interview-based scale assessing functioning in the last month in four areas: personal and social relationships, socially useful activities, self-care, and disturbing and aggressive behavior, each one with six degrees of severity characterized by specific anchor points. The total score ranges from 0 to 100, with higher scores representing better functioning.

Real-world functioning was assessed by the Specific Level of Functioning Scale (SLOF), an informant-rated measure that explores many aspects of functioning and is based on the key caregiver’s judgment on behavior and functioning of patients [64]. It consists of 43 items, divided into six different scales, and includes the following domains: physical efficiency, skills in self-care, interpersonal relationships, social acceptability, participation in community activities (e.g., shopping, using public transportation), and working abilities. Each item is rated from 1 to 5, with higher scores indicating better functioning. The SLOF has been found to be a reliable and valid instrument to assess real-world functioning with good construct validity and internal consistency: for the present study, the validated Italian version [65] was used.

### Statistical analyses

The three groups of subjects identified using the PAUSS cut-off scores were compared on demographic, cognitive, and functional measures. The distribution of scores of each considered variable was inspected for normality and homogeneity of variance in order to allow the use of parametric statistics.

Categorical variables were analyzed using Pearson’s χ^2^ tests, with results reported as percentages. Continuous variables were analyzed with general linear model analyses of variance (ANOVA).

Sociodemographic variables showing significant between-group differences were used as covariates in functional outcome comparisons; *t*-scores of cognitive domains, already corrected for gender, age, and education, were used. Post-hoc, between-groups analyses were performed accounting for multiple comparisons using Bonferroni correction.

Statistical analyses were performed using SPSS 15.0; *p*-values <0.05 (two tailed) were considered significant.

## Results

### Prevalence of autistic symptoms and sociodemographic characteristics

The mean PAUSS total score was 9.56 (SD ± 2.70, range 8–26). One hundred and ninety-two participants (56.1% of the sample) had a PAUSS score of 8 and thus were included in the “No autistic symptoms” group; 72 participants (22.1%) had a PAUSS score between 8 and 10 and were included in the “Minimal autistic symptoms” group; 78 participants (22.8%) had a PAUSS ≥ 11 and thus were included into the “Moderate autistic symptoms” group.

Comparing these three groups, no significant difference emerged regarding gender distribution, age, and education (see Table 1), so no additional covariate was introduced in between-group comparisons regarding functional outcomes.

**Table 1.** Group comparison for demographic and clinical variables

Variable	No AS Mean ± SD/% (*n*)	Minimal AS Mean ± SD/% (*n*)	Moderate AS Mean ± SD/% (*n*)	ANOVA/ Pearson χ^2^ (*p* value)	No AS vs. minimal AS (*p* value)	Minimal AS vs. moderate AS (*p* value)	No AS vs moderate AS (*p* value)
Gender							
Male	40.10 (77)	47.22 (34)	43.59 (34)	0.564	0.891	1.000	1.000
Female	59.90 (115)	52.88 (38)	56.41 (44)				
Age (years)	54.06 ± 13.09	54.53 ± 13.73	52.86 ± 14.23	0.724	1.000	1.000	1.000
Education (years)	11.22 ± 3.88	12.28 ± 4.00	11.55 ± 3.99	0.153	0.160	0.777	1.000

Abbreviation: AS, autistic symptoms.

*Note*: *Post-hoc comparisons include Bonferroni correction.*

### Between-group comparisons on cognitive performance

Significant between-group differences were observed in the Working Memory (*p* = 0.014) and Social Cognition (*p* = 0.025) domains, as well as in the global cognition composite score (*p* = 0.008). In particular, the “Moderate autistic symptoms group” showed a worse performance compared to the “No autistic symptoms” group on Working Memory (*p* = 0.012), Social Cognition (*p* = 0.020), and Global Cognition (*p* = 0.006) scores.

No difference was observed in the other investigated cognitive domains (see Table 2).

**Table 2.** Group comparison for cognitive measures

Variable	No AS Mean ± SD	Minimal AS Mean ± SD	Moderate AS Mean ± SD	ANOVA (*p* value)	No AS vs. minimal AS (*p* value)	Minimal AS vs. moderate AS (*p* value)	No AS vs. moderate AS (*p* value)
Processing speed (*t*-score)	46.33 ± 10.08	45.90 ± 9.88	43.96 ± 10.44	0.217	1.000	0.725	0.248
Attention (*t*-score)	47.18 ± 9.60	47.21 ± 11.19	44.65 ± 10.65	0.158	1.000	0.379	0.199
Working memory (*t*-score)	48.26 ± 10.33	46.44 ± 11.48	44.05 ± 11.18	0.014*	0.674	0.524	0.012*
Verbal memory (*t*-score)	48.74 ± 10.88	48.22 ± 11.09	45.64 ± 12.26	0.119	1.000	0.484	0.122
Visual memory (*t*-score)	47.69 ± 11.69	45.47 ± 13.21	44.42 ± 12.31	0.100	0.562	1.000	0.138
Problem-solving (*t*-score)	48.33 ± 10.26	47.46 ± 12.38	45.50 ± 10.15	0.146	1.000	0.793	0.151
Social cognition (*t*-score)	38.93 ± 7.18	38.18 ± 6.73	36.31 ± 7.45	0.025*	1.000	0.330	0.020*
Global cognition (composite score)	44.59 ± 10.20	43.15 ± 12.06	40.03 ± 11.25	0.008**	1.000	0.236	0.006**

Abbreviation: AS, autistic symptoms.

*Note: All cognitive measures are corrected for gender, age, education. Post-hoc comparisons include Bonferroni correction.*

* *p* < 0.05;

** *p* < 0.01.

### Between-group comparisons on functional measures

Significant between-group differences were observed for functional capacity, as measured by the UPSA-B (*p* = 0.001); for global psychosocial functioning, as measured by the PSP (*p* < 0.001); and for real-world interpersonal relationships (*p* < 0.001), participation in community activities (*p* = 0.017), and work skills (*p* = 0.006), as measured by the SLOF scale.

In particular, the “Moderate autistic symptoms group” showed a worse functional profile, with lower UPSA-B (*p* = 0.002), PSP (*p* < 0.001), SLOF Interpersonal Relationships (*p* < 0.001), SLOF Activities (*p* = 0.013), and SLOF Work (p *=* 0.019) scores as compared to the “No autistic symptoms group,” and lower PSP (*p* = 0.004), SLOF Interpersonal Relationships (*p* < 0.005), and SLOF Work (*p* = 0.010) scores as compared to the “Minimal autistic symptoms group.” The “Minimal autistic symptoms group,” compared to the “No autistic symptoms group,” showed worse global psychosocial functioning, with lower PSP scores (*p* = 0.003) (Table 3).

**Table 3.** Group comparison for functional measures

Variable	No AS Mean ± SD	Minimal AS Mean ± SD	Moderate AS Mean ± SD	ANOVA (*p* value)	No AS vs. minimal AS (*p* value)	Minimal AS vs. moderate AS (*p* value)	No AS vs. moderate AS (*p* value)
UPSA-B (functional capacity)	87.42 ± 14.71	85.00 ± 16.27	79.27 ± 18.82	0.001**	0.859	0.092	0.002**
PSP (personal and social functioning)	90.46 ± 7.39	86.28 ± 9.67	81.37 ± 12.35	<0.001**	0.003**	0.004**	<0.001**
SLOF: Physical Functioning (real-world physical efficiency)	24.48 ± 1.03	24.50 ± 0.84	24.41 ± 0.96	0.819	1.000	1.000	1.000
SLOF: Personal Care (real-world self-care skills)	34.87 ± 0.85	34.89 ± 0.43	34.74 ± 0.73	0.380	1.000	0.688	0.609
SLOF: Interpersonal Relationships (real-world interpersonal skills)	31.74 ± 4.39	31.19 ± 4.25	28.71 ± 5.86	<0.001**	1.000	0.005**	< 0.001**
SLOF: Social Acceptability (real-world social acceptability)	34.52 ± 1.05	34.31 ± 1.37	34.29 ± 1.35	0.253	0.664	0.197	0.162
SLOF: Activities (participation in community activities)	54.51 ± 1.45	54.34 ± 1.37	53.88 ± 2.17	0.017*	1.000	0.265	0.013*
SLOF: Work (real-world working skills)	27.81 ± 3.31	28.24 ± 3.21	26.49 ± 4.53	0.006**	1.000	0.010*	0.019*

Abbreviations: AS, autistic symptoms; PSP, personal and social performance scale; SLOF, specific level of functioning scale; UPSA-B, UCSD performance-based skills assessment–brief version.

*Note: Post-hoc comparisons include Bonferroni correction.*

* *p* < 0.05;

** *p* < 0.01.

## Discussion

Several interesting results concerning both the prevalence and the correlates of autistic symptoms emerged from the analyses.

Autistic characteristics can be considered a continuum of features in the general population, and therefore subthreshold levels of autistic symptoms can be observed in nonclinical samples [66–69]. In fact, while the global prevalence of ASD diagnosis can be attested between 1.6% and 2.6% [70–73], a recent study [74] has highlighted that 17.6% of healthy controls show significant subthreshold autistic features measured with a dedicated assessment tool [66, 75, 76].

However, 44.9% of the present sample showed autistic symptoms, that is, 22.8% were of moderate severity, which is a level commonly observed in the majority of people living with SSD [17, 28, 48].

The prevalence of autistic symptoms in this sample is however consistently lower than that observed using the same instrument in large samples of people with SSD: in the NIRP sample, 73.8% of participants showed moderate autistic symptoms and 20.1% severe autistic symptoms [28]. Another recent study conducted in China using the PAUSS did not include data regarding moderate autistic symptoms, but reported that 18.6% had severe autistic features [77].

This finding suggests that autistic features could be more frequent in first-degree relatives of individuals diagnosed with SSD than in the general population, but less frequent and less severe than those observed in people living with SSD.

In participants with more severe autistic symptoms, a worse cognitive performance was observed, both in the Global Cognition composite index and in Working Memory and Social Cognition domains. This finding is line with those observed in people with SSD and confirms the relationship between autistic symptoms and worse cognitive performance, which is particularly important regarding social cognition abilities [8, 9, 28, 78, 79].

Participants with more severe levels of ASD symptoms also showed worse functional capacity and worse psychosocial functioning, particularly in areas where social abilities are more relevant, such as real-world interpersonal relationships, community activities, and work outcomes.

This is another expected finding, which is again in line with those observed in people living with SSD [26, 28, 32, 80]. Participants with minimal autistic symptoms did not show worse real-world functional outcomes compared to those without autistic symptoms; however, they showed significantly reduced personal and social functioning as measured by the PSP. In this regard, minimal autistic symptoms might not be as clinically relevant as moderate or more severe autistic features, but could still deserve scientific attention and observation.

Taken together, all these findings suggest that autistic features have a similar role in people living with SSD and in their first-degree relatives and confirm the similarities in cognitive and functional impairment patterns observed in these two populations [42, 44]. However, while autistic symptoms may also have a protective effect in people with SSD, limiting negative impact of high levels of positive symptoms on functional outcomes [32–34], first-degree relatives of people living with SSD typically do not present positive symptoms, so autistic symptoms may be even more detrimental in this population.

Detecting autistic features in first-degree relatives, therefore, could also represent a useful feature in clinical practice. According to the results of this study, the presence of autistic symptoms could represent a marker of functional impairment in first-degree relatives of people living with SSD. This could be of clinical interest both because first-degree relatives of patients with SSD often have a contact with mental health services either for their diagnosed relative or for conditions of their own and because assessing autistic symptoms, particularly with the PAUSS, represents a much faster assessment than a complete evaluation of functional capacity and functional outcomes. In fact, this may allow to easily identify individuals who, even without a clear diagnosis of SSD, might show relevant levels of functional impairment and may particularly benefit, alongside their diagnosed relatives, from evidence-based psychosocial interventions targeting cognitive and functional outcomes [81–86].

The present study shows some remarkable strengths.

To the best of our knowledge, it represents the first comprehensive assessment of the impact of autistic symptoms in first-degree relatives of people living with SSD on both cognitive and functional outcomes.

The inclusion of a large sample of participants, combined with the use of a wide panel of well-validated assessment tools, contributes to the validity and reproducibility of the observed results.

However, the present study has also some limitations.

The PAUSS was designed specifically to investigate the severity of autistic symptoms and the correlates of the autistic phenotype in SSD: the present study included first-degree relatives of people living with schizophrenia, which can be considered an intermediate phenotype.

In this perspective, other instruments, designed to assess ASD features in the general population, such as the Autism Diagnostic Observation Schedule [87] or the Adult Autism Subthreshold Spectrum [66], may be more adequate to assess and differentiate subjects with minimal levels of autistic features. However, specific instruments aimed to assess autistic characteristics usually require dedicated training and longer administration times compared to the PAUSS [52]. The present study did not include a sample of participants with high levels of autistic symptoms (PAUSS > 30). Again, this might be due to the included sample, composed exclusively of unaffected relatives without psychiatric comorbidities, but it might have limited the potentiality of the PAUSS scale, which reliably allows to identify people living with SSD showing a clear autistic phenotype [52]. Moreover, the present study did not include a sample of participants drawn from the general population: for this reason, it was not possible to directly compare the prevalence, severity, and the correlates of autistic symptoms measured with the PAUSS in our sample with those of healthy controls without a diagnosis of any mental disorder who were not first-degree relatives of people living with SSD. Finally, while the PAUSS has been extensively validated [27, 48, 52] and employed in several different international studies with large samples of people with SSD [26, 28, 32, 77], more data regarding its clinical specificity, in particular its long-term stability, is currently required [50].

However, by using the PAUSS we were able to highlight significant cognitive and functional correlates in the present sample, particularly in subjects with relatively higher levels of autistic symptoms.

In conclusion, the results of the present study show that autistic symptoms in first-degree relatives of people with SSD are correlated with lower levels of cognitive performance and real-life functioning also in this population and may represent a diagnostic and treatment target of considerable scientific and clinical interest in both populations.

Future studies should focus on further assessing the role of autistic symptoms as a predictor and modulator of treatment response for both psychosocial interventions and pharmacological therapies in order to better devise personalized treatment programs that are more effective and useful for both people living with SSD and their families.

### Appendix

Members of the Italian Network for Research on Psychoses involved in this study include Alessandro Galluzzo, Anna Ceraso, Jacopo Lisoni (University of Brescia); Enrico D’Ambrosio, Ileana Andriola, Pierluigi Selvaggi (University of Bari); Federica Pinna, Luca Marras, Michele Muscas (University of Cagliari); Giuseppe Piegari, Francesco Brando, Giulia Maria Giordano, Pasquale Pezzella (University of Campania “Luigi Vanvitelli”, Naples); Carmen Concerto, Alessandro Rodolico, Maria Salvina Signorelli (University of Catania); Mauro Pettorruso, Stefania Chiappini, Giacomo d’Andrea (University of Chieti); Stefano Pallanti (University of Firenze); Mario Altamura, Laura De Masi, Ivana Leccisotti (University of Foggia); Pietro Calcagno, Anna Bovio, Juxhin Bode (University of Genoa); Lorena Campoli, Luca Bonanni, Arianna Di Berardo, Laura Giusti, Silvia Mammarella, Anna Salza (University of L’Aquila); Matteo Marcatili, Oscar Fusi (University of Milan); Carla Gramaglia, Eleonora Gambaro, Valentina Zanoli (University of Eastern Piedmont, Novara); Angela Favaro, Paolo Meneguzzo, Enrico Collantoni (University of Padua); Matteo Tonna, Davide Fausto Borelli, Francesca Magnani (University of Parma); Barbara Carpita, Ivan Mirko Cremone, Giulia Amatori (University of Pisa); Giammarco Cascino, Giulio Corrivetti, Gianfranco Del Buono (Department of Mental Health, Salerno); Claudio Brasso, Gianluca Colli, Rodolfo Sgro (University of Turin); Antonino Buzzanca, Tommaso Accinni, Fabio di Fabio, Anna Comparelli, Isabella Berardelli, Denise Erbuto (Sapienza University of Rome); Andrea Fagiolini, Arianna Goracci, Simone Bolognesi (University of Siena); Cinzia Niolu, Giorgio Di Lorenzo, Emanuela Bianciardi (Tor Vergata University of Rome).
